# Cranioplasty After Removal of a Meningioma With Skull Invasion: A Technical Case Report

**DOI:** 10.7759/cureus.84536

**Published:** 2025-05-21

**Authors:** Taigen Sase, Homare Nakamura, Gaku Hidaka, Kiyotaka Wakatsuki, Hidetoshi Murata

**Affiliations:** 1 Department of Neurosurgery, St. Marianna University School of Medicine, Yokohama Seibu Hospital, Yokohama, JPN; 2 Department of Neurosurgery, St. Marianna University School of Medicine, Yokohama, JPN

**Keywords:** calcium phosphate bone paste, cranioplasty, meningioma, skull bone strengthening, skull invasion

## Abstract

This case report describes a novel cranioplasty technique using calcium phosphate paste. The patient was a man in his 50s with a convexity meningioma with skull invasion extending to the diploic layer. Craniotomy was performed, and the area of skull invasion was removed. Afterward, the skull defect was filled with calcium phosphate bone paste to maximize bone strength. To prevent the bone paste from falling off postoperatively, the area of skull invasion was removed more widely from the diploic plate side than from the inner side. This method may be a useful option for strengthening the skull in cases of cranioplasty for meningiomas with skull invasion.

## Introduction

Meningiomas are among the most common brain tumors, and these occasionally invade the skull. Hyperostosis of the cranium is reported in 4.5-17% of cases of intracranial meningioma [1,2], and in such cases, invasion occurs at a high rate of 96% [3]. Skull invasion is associated with an increased risk of recurrence and mortality [4]. Accordingly, surgery aims to completely remove the tumor, including the attached dura mater and the part that has invaded the skull. The method of skull reconstruction after removing invasive tumors depends on the degree of tumor infiltration and removal. In cases of mild hyperostosis, the visible hyperostotic skull bone can be drilled from the inner layer before replacing the craniotomy bone flap [5]. Craniectomy surgery with meningioma resection may be anticipated if the tumor traverses partly or completely through the associated bone based on imaging. Alloplastic cranioplasties can be performed with a variety of materials, including titanium mesh or plate, ceramics, acrylics, and so on [6].

In the present case, we removed a convexity meningioma with skull invasion extending to the inner and diploe layer. After performing craniotomy and removing the skull-invaded area except the outer layer, the defects were filled with calcium phosphate bone paste (BIOPEX®-R ADVANCE; HOYA Technosurgical Corporation, Tokyo, Japan), and skull reconstruction was performed from the inside. We report the ingenuity of this technical case.

## Case presentation

The patient was a man in his 50s who worked as a truck driver (involving helmet-wearing duties) with a history of hypertension and dyslipidemia. Six years ago, a head magnetic resonance imaging (MRI) incidentally revealed a meningioma, which was kept under observation. The tumor increased in size over time, and the patient was eventually referred to our department for treatment in July 2023. After various examinations, surgical management was chosen to remove the growing tumor. 

**Figure 1:**
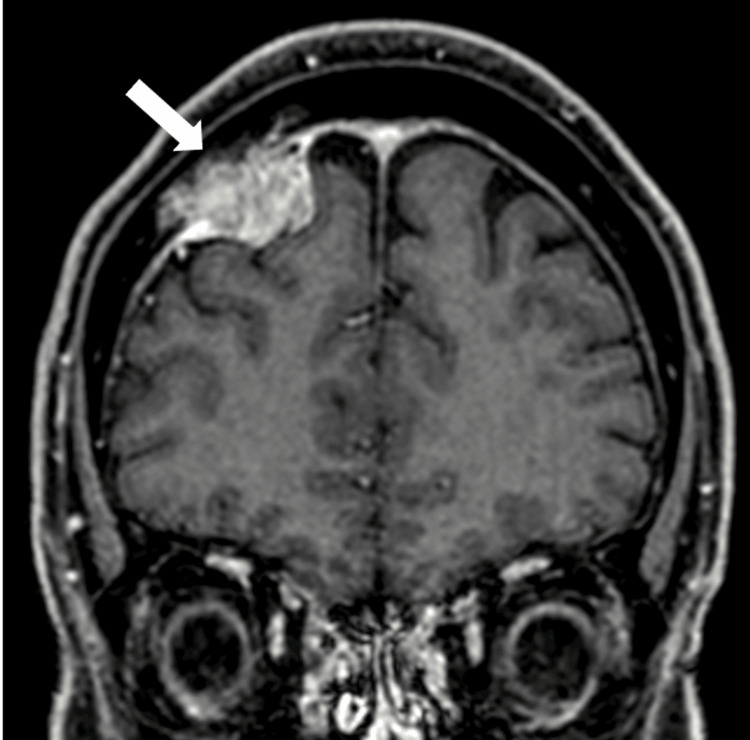
Preoperative head contrast MRI revealing a meningioma with tumor invasion into the diploic layer in the right frontal skull bone (white arrow)

Contrast MRI revealed tumor infiltration into the right frontal bone, extending to the diploic layer (Figure 1). After tumor-feeding artery embolization the day prior, craniotomy was performed under general anesthesia. The head of the patient was fixed using three pins. A navigation system was used to determine the craniotomy site. Bicoronal incisions were made on the scalp, then bilateral frontal craniotomies were performed. The tumor that had spread extradurally was removed. Afterward, the right side of the dura mater was incised, and the intradural tumor was removed under a microscope. 

**Figure 2:**
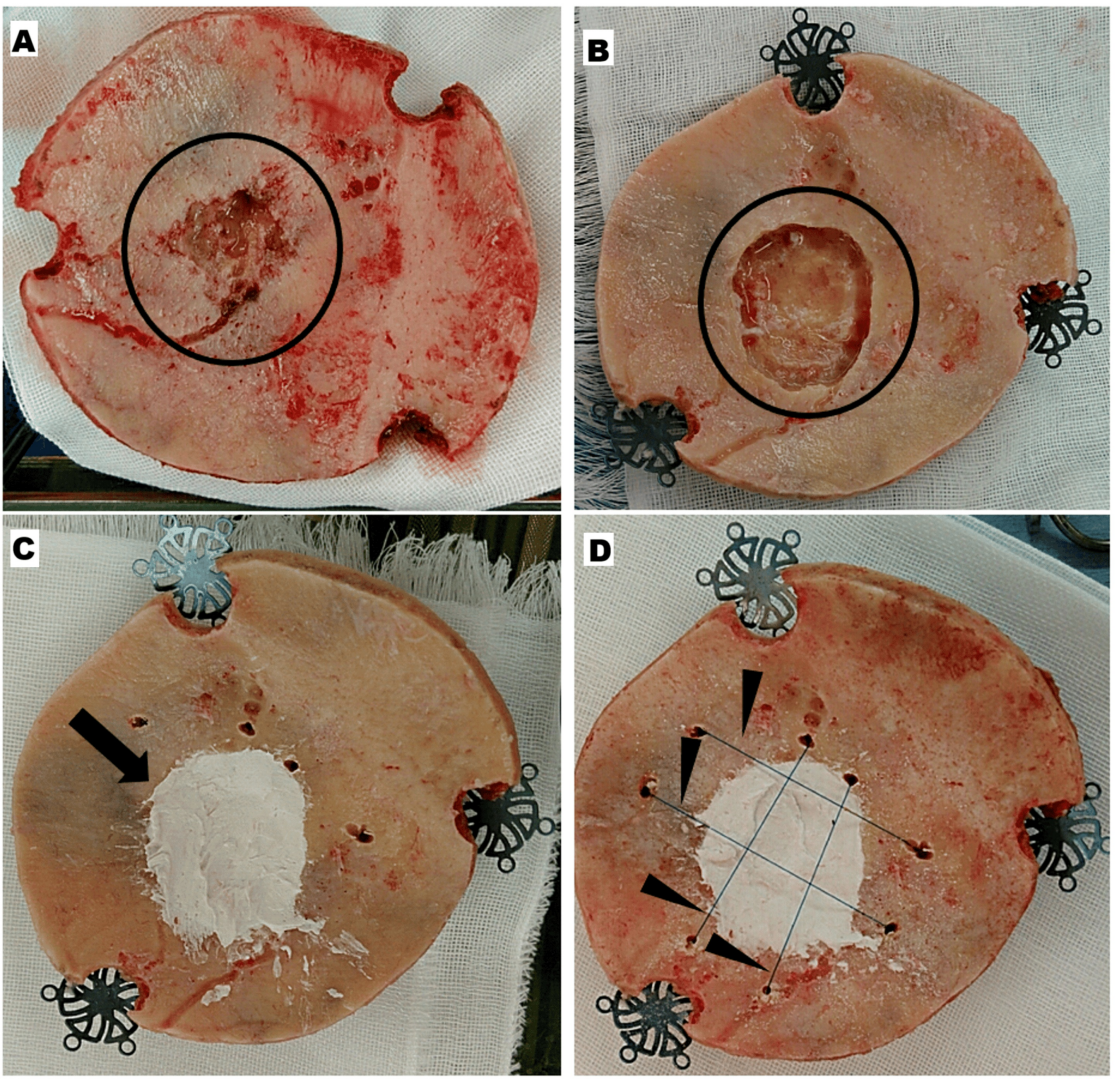
Intraoperative photographs (A) Tumor invasion was observed on the inner surface of the craniotomy skull bone (black circle); (B) The craniotomy skull bone was removed to cover the area of tumor invasion (black circle), while leaving the outer plate; (C) The defect was filled with calcium phosphate bone paste (black arrow); (D) Multiple holes were made in the normal bone surrounding the bone paste, and nylon threads were used to secure it (black arrowheads).

The attached dura mater was resected and replaced with a synthetic artificial dura mater. Subsequently, a fibrinogen-added factor XIII preparation was sprayed onto the dura mater and artificial dura mater surfaces. However, some bone defects due to tumor infiltration were observed on the inner surface of the craniotomy-treated bone (Figure 2A). Bone was removed from the area of skull invasion using a ball-head cutting bar, such that the tumor, which invaded the inner plate and diploic layer, could be completely removed while leaving the outer plate intact (Figure 2B). The thin craniotomy bone after drilling, only the outer plate, felt weak to the touch. To maximally reinforce the strength of the bone defect, it was filled from the inside with calcium phosphate bone paste (BIOPEX-R ADVANCE) formed into the shape of the bone (Figure 2C). 

**Figure 3:**
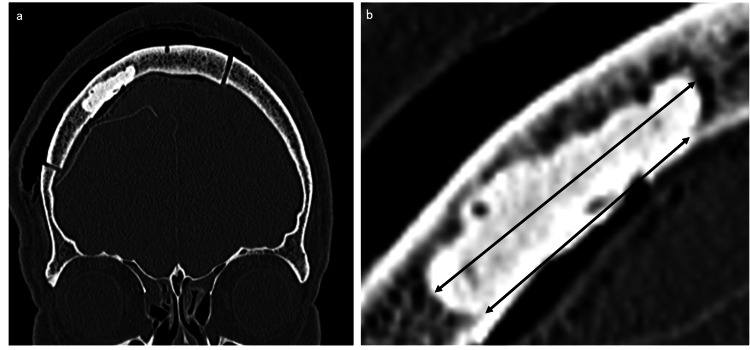
Postoperative CT To prevent the bone paste from falling off postoperatively, the skull bone was removed more widely on the diploic plate side than on the inner side (a: coronal imaging of postoperative CT, and b: enlarged view). The volume of bone defect was 3.77 cc (Ziostation2 version 2.9.5.1, Ziosoft, Tokyo).

To prevent the bone paste from falling off after surgery, the area of skull invasion was removed more widely on the diploic layer side than on the inner plate side (Figure 3). Additionally, multiple holes were made in the normal bone surrounding the bone paste plasty, and nylon threads were used to secure the bone paste around the area (Figure 2D). After allowing the bone paste to harden for approximately 10 minutes, the craniotomy bone was fixed using a titanium plate and returned to the skull. Lastly, the wound was sutured layer by layer. Computed tomography (CT) performed immediately after surgery confirmed that the bone paste adequately covered the defect. The pathological finding of the intracranial tumor was atypical meningioma, with an MIB-1 index of approximately 5%. Similar tumor cells were also identified in the pathological findings of the skull invasion area. 

**Figure 4:**
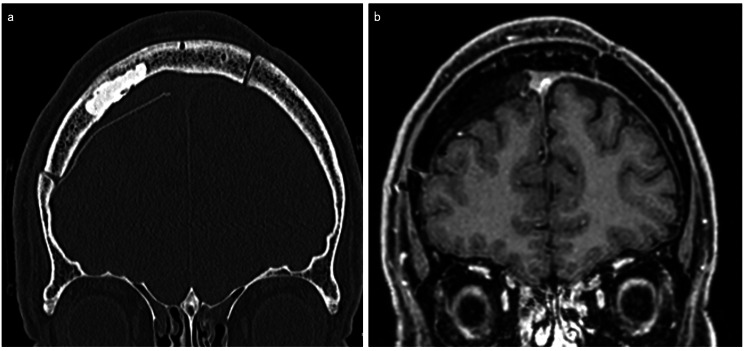
Postoperative CT scan and contrast MRI (a) CT scan taken nine months after surgery showed no sign of bone paste falling off; (b) Contrast MRI performed one year after surgery showed no recurrence in the cranioplasty area

The postoperative course was uneventful, and the patient was discharged on the seventh day after surgery. In the first six-month follow-up, there were no signs of the bone paste falling off and tumor recurrence on CT and MRI. In addition, during the same period, there were no foreign body reactions, infections, or cosmetic deformities. A CT scan performed nine months postoperatively showed no signs of the bone paste falling off (Figure 4a), and contrast MRI performed one year postoperatively showed no recurrence (Figure 4b), including the area of bone infiltration. The patient was noted to have good progress at one year and six months postoperatively. In the follow-up observations, there were no complications, including foreign body reactions or infections.

## Discussion

This case report described a novel cranioplasty technique using calcium phosphate bone paste. After removing a convexity meningioma with skull invasion extending down to the inner plate and diploic layer except the outer plate, the skull defect was filled with bone paste, forming a craniotomy skull bone from the inside as a result.

Meningiomas occasionally invade the skull, and the surgical management of such cases remains widely debated. The extent of meningioma resection is a prognostic factor for recurrence and survival [7,8], and craniectomy is often required in cases with skull invasion. To achieve Simpson classification I resection, the meningioma is often completely removed, and the attached dura mater and areas of skull invasion are removed to minimize recurrence [7].

The method of plasty for skull defects is determined by the degree of tumor invasion and resection. Complete resection of the skull infiltration area, including the area of invasion, is the most effective method of craniectomy. In such cases, the craniotomy area must be determined in advance to create a custom-made artificial bone to be used for skull plasty. Alternatively, the skull defect can be protected with a mesh plate or a similar material. The in situ cranioplasty technique using titanium mesh and methyl methacrylate enables a single-step surgical procedure that can excise a large skull tumor and create a structurally sound and cosmetically acceptable cranioplasty [9,10]. If only the invaded areas of the skull are removed, the plasty method varies depending on whether the resection reaches the outer plate and creates a skull bone defect during craniotomy. For small skull bone defects, one option is to avoid skull defect reformation, especially in malignant tumors of the skull and scalp [11]. From a cosmetic standpoint, small defects can be filled with a titanium plate or calcium phosphate paste. If the outer plate is preserved while removing the area of skull infiltration, the most common procedure involves returning the craniotomy bone.

The outer and inner plates of the cortical bone both contribute to the strength of the skull. Removing the inner plate of the skull halves its bone strength, as described by Emura et al. in 10 cases of autologous split calvarium cranioplasty for a pediatric skull defect [12]. Melvin JW’s fracture classification type 1 is prone to occur in small areas (< 1 in2) affected by high-speed impacts, specifically in the frontal and parietal bones, with the development of the diploic layer [13]. Additionally, the thinner parts of the skull are weaker, and holes are more likely to cause fractures due to stress concentration [14]. Thus, if the inner plate and diploe layers were removed, only the thinned outer plate would remain, which would certainly be weaker, but this is usually not a major problem in daily life. However, if the patient has special considerations, such as a job that requires caution against falling objects, strengthening the skull bone with defects may be considered.

Considering the current patient’s occupational background, we decided to perform bone plasty based on the internal shape and strength. The defects were filled with bone paste, although its strength is not equal to that of normal skull bone. To prevent the bone paste from falling off postoperatively, the area of skull invasion was removed more extensively on the diploic layer side than on the inner plate side (Figure 3). Additionally, multiple holes were made in the normal bone surrounding the bone paste, and nylon threads were used to tie the bone paste around the area (Figure 2D).

Calcium phosphate bone paste is an α-tricalcium phosphate-based bioactive cement that changes to hydroxyapatite through hydration in vivo, thus having an affinity with bone and excellent tissue compatibility. The viscosity of the paste can be adjusted to the desired consistency by altering the mixing ratio of the powder and liquid. When used on a craniotomy bone, as in this case, the defect area can be filled with calcium phosphate bone paste in a clean field outside the surgical field. Afterward, the craniotomy bone can be returned to the skull after the required hardening time, which was seven minutes in this case for the calcium phosphate bone paste. In terms of compressive strength, this is approximately 80-160 MPa for regular cortical bone, while the calcium phosphate bone paste should reach 80 MPa within 24 hours. Thus, filling the defective inner plate and diploid layer with bone paste can strengthen the skull. Another advantage of using calcium phosphate bone paste is its long-term bone-healing effects, although this effect is not immediate. The measures taken to help adhere the bone paste were effective in this regard. 

In the follow-up observations, there were no foreign body reactions or absorption changes, even one year and six months after surgery. However, the limitation of this technique is the unknown long-term behavior of the paste under external stress (e.g., helmet-wearing patient).

## Conclusions

A convexity meningioma with skull bone invasion was removed, and the resulting defect of the inner plate and diploic layer was filled with calcium phosphate bone paste. The bone was plastied from the inside, aiming to reinforce the area with as much strength as possible. There are two key points to the technique of cranioplasty from the inside using bone paste. The first key point is to prevent the bone paste from falling off after surgery, the area of skull invasion was needed to remove more widely on the diploic layer side than on the inner plate side. The second point is to drill multiple holes in the normal bone surrounding the bone paste plasty and use nylon threads to secure the bone paste around the area. This novel cranioplasty technique using calcium phosphate paste was beneficial, and can be used to improve bone strength in similar cases.
